# Early prediction of pathologic response to neoadjuvant treatment of breast cancer: use of a cell-loss metric based on serum thymidine kinase 1 and tumour volume

**DOI:** 10.1186/s12885-020-06925-y

**Published:** 2020-05-18

**Authors:** Bernhard Tribukait

**Affiliations:** 1 Department of Oncology-Pathology, Karolinska Institute and University Hospital Solna, Stockholm, Sweden; 2 Cancer Centrum Karolinska, CCK, Plan 00, Visionsgatan 56, Karolinska Universitetssjukhuset, Solna, 17164 Stockholm, Sweden

**Keywords:** Circulating thymidine kinase 1, Cell-loss, Biomarker, Treatment response, Breast cancer

## Abstract

**Background:**

After neoadjuvant chemotherapy of breast cancer pathologic complete response (pCR) indicates a favorable prognosis. Among non-selected patients, pCR is, however, achieved in only 10–30%. Early evaluation of tumour response to treatment would facilitate individualized therapy, with ineffective chemotherapy interrupted or changed. The methodology for this purpose is still limited. Tumour imaging and analysis of macromolecules, released from disrupted tumour cells, are principal alternatives.

**Objective:**

To investigate whether a metric of cell-loss, defined as the ratio between serum concentration of thymidine kinase1 (sTK1, ng x ml^− 1^) and tumour volume, can be used for early prediction of pathologic response.

**Methods:**

One hunred four women with localized breast cancer received neoadjuvant epirubicin/docetaxel in 6 cycles, supplemented with bevacizumab in cycles 3–6. The cell-loss metric was established at baseline (*n* = 104), 48 h after cycle 2 (n = 104) and prior to cycle 2 (*n* = 57). The performance of the metric was evaluated by association with pathologic tumour response at surgery 4 months later.

**Results:**

Treatment caused a rise in sTK1, a reduction in tumour volume and a marked increase in the cell-loss metric. Patients were subdivided into quartiles according to the baseline cell-loss metric. For these groups, baseline means were 0.0016, 0.0042, 0.0062, 0.0178 units. After subtraction of baselines, means for the quartiles 48 h after treatment 2 were 0.002, 0.011, 0.030 and 0.357 units. pCR was achieved in 24/104, their distribution in the quartiles (11, 11, 23 and 46%) differed significantly (*p* = 0.01). In 80 patients with remaining tumour, tumour size was inversely related to the metric (*p* = 0.002). In 57 patients studied before treatment 2, positive and negative predictive values of the metric were 77.8 and 83.3%, compared to 40.5 and 88.7% 48 h after treatment 2.

**Conclusion:**

A cell-loss metric, based on serum levels of TK1, released from disrupted tumour cells, and tumour volume, reveal tumour response early during neoadjuvant treatment. The metric reflect tumour properties that differ greatly between patients and determine the sensitivity to cytotoxic treatment. The findings point to the significance of cell loss for tumour growth rate. The metric should be considered in personalized oncology and in the evaluation of new therapeutic modalities.

**Trial registration:**

PROMIX (Clinical Trials.govNCT000957125).

## Background

Neoadjuvant chemotherapy (NACT) has become a treatment option for patients with early stage breast cancer (BC) [1–4]. The acceptance of NACT in routine treatment is based on long-term follow-up of large cohorts of patients, sub-grouped according to tumour characteristics and undergoing equal programmes of neoadjuvant or adjuvant chemotherapy [5, 6]. Clinical benefits of NACT are related to down-staging of the tumour, which reduces the extent of surgery and permits a higher rate of breast-conserving surgery [1, 3, 6]. The gold standard for evaluating the effect of NACT is pathologic response established at surgery. Thus, at this point in time individual tumour characteristics are revealed which are important when considering prognosis and further treatment. Pathologic complete response (pCR) has been found to be associated with a favorable long-term outcome [1–6].

NACT provides valuable opportunities also in the perspective of clinical research. With pCR as endpoint, the effectiveness of new treatments may be established without several years of follow-up, as would be the case with disease-free or overall survival. For instance, pertuzumab for treatment of high-risk early stage BC received, therefore, an accelerated FDA-approval [7]. Likewise, the NACT setting facilitates the elucidation of biochemical mechanisms of cytotoxic or cytostatic effects. A related issue is the heterogeneity of BC and the fact that the response to therapy may differ greatly between patients. The common anthracycline/taxane treatment of non-selected patients results in pCR in only 10–30% of cases [2, 5, 6, 8]. Accordingly, in 70–90% of patients chemotherapy fails to eradicate the primary tumour. These differences in response indicate heterogeneity of BC beyond the traditional classification. Gene expression analyses have revealed sub-types of tumours, differing in oncogenic signalling pathways, and these constitute potential targets of new therapies [9]. Because of cross-talk between such pathways optimal therapy might require combinations of various pathway inhibitors [10].

The growing insight into the diversity of BC has generated an increasing demand for methods that may facilitate, in the individual patient, early evaluation of the response to NACT. Identification of tumours with poor response would permit a switch in chemotherapy or motivate proceeding with immediate surgery - and suffering due to fruitless cytotoxicity could be avoided. Hence, individualized or response-guided therapy has become a prominent subject in present oncology. Nevertheless, a general obstacle is that tumour sensitivity to drugs can only be established in a minority of patients.

Several available methods have the potential of predicting pathologic tumour response during therapy: (i) measurement of changes in tumour size, (ii) estimation of tumour metabolism using radioactive tracer uptake, and (iii) measurements of the concentration of macromolecules released from disrupted tumour cells into the blood circulation. Most frequently used are anatomical measurements of tumour size, and criteria of response are defined in the Response Evaluation Criteria in Solid Tumors (RECIST) [11]. For tracer studies, like PET with 18F-fluorodeoxyglucose or deoxy*-*18F-fluorothymidine, response assessment criteria have still not been established [12]. A general problem in the assessment of tumour response via the release of macromolecules is related to the fact that cytotoxic substances do not exert their effect specifically in tumour tissue; usually the quantity of affected normal tissues greatly exceeds that of the tumour. For instance, although mutations in circulating DNA fragments make them specific for the tumour, the much higher level of non-tumour DNA may interfere with the measurement of circulating tumour DNA. Hence, circulating tumour DNA has mainly been used in the study of cancer-associated mutations or for monitoring of clonal evolution and development of resistance to therapy [13, 14]. For unspecific macromolecules, an origin in the tumour may be established via the association between their serum concentrations and tumour properties like volume, growth rate, or response to therapy.

In the present study the release into the blood circulation of thymidine kinase1 (TK1) during chemotherapy has been used to create a measure of cell loss. The cytoplasmatic TK1 is a key enzyme in DNA synthesis, catalysing thymidine into deoxythymidine monophosphate from extracellular sources via the salvage pathway. TK1 is cell cycle dependent: being undetectable in G0/G1, its concentration increases at the G1/S-phase border and reaches peak values during S-phase/G2. It is finally degraded in mitosis by ubiquitination [15, 16]. In connection with death of proliferating cells, TK1 is released into blood; hence increased serum concentrations (sTK1) have been found in patients with malignancies, including BC [17, 18]. Serial measurements of sTK1 in BC patients undergoing NACT have revealed a close association between changes in sTK1 during chemotherapy and tumour response, established at surgery as endpoint [19]. This association became more evident if sTK1 was related to the tumour volume early during treatment.

### Aim of the study

The aim of the present study was to investigate the usefulness of a measure of cell loss, defined as the ratio between sTK1 and tumour volume. We hypothesized that, whereas sTK1 is most likely dependent on tumour volume, the cell-loss metric would be more closely related to functional properties of the tumour, i.e. the occurrence of cell loss in undisturbed tumour growth or the enhanced cell loss during chemotherapy. To this end, in BC patients the cell-loss metric, established prior to NACT and in conjunction with the 2nd cycle of therapy, was related to pathologic response at surgery as objective end-point 4 months after initiation of chemotherapy. An association of the cell-loss metric with pathologic response would also confirm the tumour specificity of sTK1, thereby highlighting the issue of possible pathways for elimination of disrupted tumour cells during chemotherapy.

## Methods

### Study design and treatment

This study is part of the neoadjuvant, multicentre single-arm Phase II clinical trial, PROMIX (Clinical Trials.govNCT000957125). The study was approved by the Ethics Committee at Karolinska University Hospital, 2007/1529–31/2, and informed written consent was obtained from all patients. The inclusion criteria and treatment protocol are fully described elsewhere [20]. Briefly, between 2008 and 2011, 150 women with primary locally advanced but operable HER2-negative breast cancer with or without regional lymph node metastases were enrolled. Other inclusion criteria were: age ≥ 18, adequate bone marrow, renal, hepatic and cardiac functions and no uncontrolled medical or psychiatric disorders. Main exclusion criteria were distant metastases, other malignancies, pregnancy or lactation.

The patients were scheduled for 6 cycles of epirubicin and docetaxel (75 mg/m^2^ i.v. each) every 3 weeks, and in the absence of clinical complete response (cCR) after the 2nd cycle, for the addition of bevacizumab (15 mg/kg i.v.) on day 1 of cycles 3–6. Within 3 weeks after completing chemotherapy the patients underwent surgery and were eventually further treated in accordance with the Swedish national guidelines.

The present ad-hoc study comprised 104 women from whom we had complete sets of data on sTK1 and tumour volume at baseline and 48 h after the 2nd cycle of chemotherapy together with assessment of the pathological status at surgery after 6 cycles of chemotherapy (see flow chart, additional material). For 57 of the patients, sTK1 and tumour volume had also been obtained prior to the 2nd cycle; these data were used for comparisons with the data 48 h after the 2nd cycle but were not included in the overall analysis.

### Data collection

Serum thymidine kinase1 concentration: For collection of serum, venous blood was drawn in 5 ml plastic tubes. The tubes were inverted 10 times, the blood sample was allowed to clot for 30–60 min and centrifuged for 10 min at 1500 RCF = g at room temperature. After transfer of serum to a new tube, it was centrifuged at 3000 RCF = g for 10 min at room temperature, and transferred to new tubes in aliquots of 0.5 ml to be immediately frozen at -20 °C or -80 °C for storage at -80 °C until analysis.

The concentration of TK1 protein in serum was measured at the Department of Anatomy, Physiology and Biochemistry, Swedish University of Agricultural Sciences, Uppsala, Sweden, with the new sandwich TK210 ELISA, produced by AroCell AB, Uppsala, Sweden. This test is based on two monoclonal antibodies against the C-terminal region of the TK1 protein and was performed in accordance with the manufacturer’s instruction (www.arocell.com). Samples were blinded with respect to patient identity, clinical data or tumour pathology.

#### Clinical tumour volume

The tumours were considered to be spherical and their volumes (cm^3^) were calculated by assessment of the largest diameter from caliper examinations, mammography and/or ultrasound. Tumour volume was measured at baseline and after the 2nd cycle of therapy.

#### Other factors

The local pathologists did immunohistochemical analyses of biopsied tumour material before chemotherapy. To distinguish luminal A from luminal B, a Ki67/Mib1 labelling index of 20% was assumed. Estrogen and progesterone receptor status was classified as positive if at least 10% of the cells were stained. After closure of the trial the tumours were subsequently also genetically classified by the PAM50 gene signature [20] and combined into three categories of luminal A, luminal B and basal.

#### Pathological status at surgery

Histologic response was evaluated by the local pathologists and discussed at clinical-onco-pathologic conferences. Pathologic complete response (pCR) was defined as absence of invasive cancer in the breast; residual non-invasive DCIS was allowed. Remaining cancers were classified according to size into pT1-pT3, and volume of the tumours was calculated from their largest diameter. Regional lymph node status was not taken in account for pCR because response to therapy could not be assessed during therapy.

### Statistical analysis

To obtain an estimate of the proportion of proliferating tumour cells being disrupted due to chemotherapy, the value of sTK1 48 h after the 2nd cycle was divided by the measure of tumour volume obtained between the 2nd and 3rd cycle. From this cell-loss metric, the baseline metric was subtracted. The cell-loss metric at baseline reflects the spontaneous disruption of proliferating tumour cells together with the background release from a minority of normal cells. Based on the cell-loss metric at baseline, the 104 patients were divided into quartiles. For each quartile the percentage of pCR was calculated. Additionally, for a subgroup of 57 patients the cell-loss metric, corrected for baseline, was also established before cycle 2. Possible differences in percentages between groups were examined with Fisher’s exact test and for absolute changes Wilcoxon test. A two-sided *p*-value below 0.05 was considered as indicating statistical significance. Concerning baseline characteristics and pathological outcome, analysis of variance was applied to examine the associations. Receiver operating characteristic (ROC) curves was used to assess the discriminating power for differentiating pCR from patients with incomplete response. All analyses were done using the statistical software Statistical Analysis Software, SAS, Cary, NC. USA.

## Results

In the flow chart (additional material Flow chart) the reason for missing information and excluding patients from the analyses are accounted for. Table 1 shows baseline demographic data in the four quartile groups of patients. Tumour volume and, hence, stage and cell-loss metric were the only baseline characteristics in which statistically significant differences were found between the four quartiles.

**Table 1:** Characteristics of patients, tumours and cell-loss

Variable	Statistics	Total	Quartile 1	Quartile 2	Quartile 3	Quartile 4
Age at	n	104	26	26	26	26
registration	Mean (Std)	50.0 (9.8)	49.2 (8.2)	52.4 (9.3)	51.0 (10.6)	47.4 (10.9)
	Median (min;max)	50.0 (27.8;69.2)	50.5 (30.0;61.4)	50.3 (35.3;66.3)	52.5 (33.1;69.2)	47.4 (27.8;65.4)
	Q1, Q3 (IQR)*	41.3, 58.4 (17.0)	44.2, 56.5 (12.4)	46.0, 61.7 (15.7)	40.6, 58.2 (17.6)	38.6, 58.8 (20.2)
Menopause	Post: n (%)	42 (40.4)	9 (34.6)	13 (50.0)	12 (46.2)	18 (69.2)
	Pre: n (%)	62 (59.6)	17 (65.4)	13 (50.0)	14 (53.8)	11 (42.3)
Stage	1: n (%)	3 (2.9)	0 (0)	0 (0)	0 (0)	3 (11.5)
	2: n (%)	37 (35.6)	0 (0)	5 (19.2)	14 (53.8)	18 (69.2)
	3: n (%)	64 (61.5)	26 (100)	21 (80.8)	12 (46.2)	5 (19.2)
Tumour	n	104	26	26	26	26
volume, cm^3^	Mean (Std)	193 (384)	474 (681)	139 (82)	79 (68)	81 (143)
	Median	113	253	113	65	33
	(min;max)	(4;3052)	(87;3052)	(17;381)	(14;321)	(4;696)
	Q1, Q3	33, 180	113, 435	87, 179	33, 113	14, 65
	(IQR) *	(146)	(322)	(92)	(79)	(51)
sTK1 ng/ml	n	104	26	26	26	26
	Mean (Std)	0.34 (0.18)	0.32 (0.12)	0.35 (0.17)	0.30 (0.13)	0.40 (0.25)
	Median (min;max)	0.30 (0.1;1.29)	0.30 (0.12;0.57)	0.39 (0.1;0.93)	0.28 (0.11;0.57)	0.28 (0.15;1.29)
	Q1, Q3	0.23, 0.44	0.24, 0.42	0.23, 0.42	0.18, 0.40	0.24, 0.51
	(IQR) *	(0.208)	(0.172)	(0.195)	(0.225)	(0.36)
Cell-loss	n	104	26	26	26	26
Metric, units						
	Mean (Std)	0.0074 (0.0125)	0.0016 (0.0014)	0.0042 (0.0063)	0.0062 (0.0054)	0.0178 (0.0203)
	Median	0.0033	0.0011	0.0024	0.0045	0.0107
	(min;max)	(0.0001;0.0693)	(0.0001;0.0050)	(0.0006;0.0326)	(0.0011;0.0241)	(0.0004;0.0693)
	Q1, Q3	0.0016, 0.0065	0.0005, 0.0023	0.0016, 0.0041	0.0028, 0.0072	0.0053, 0.0195
	(IQR)*	(0.0049)	(0.0018)	(0.0025)	(0.0043)	(0.0142)
Histological type	Ductal: n (%)	73 (70.2)	18 (69.3)	16 (61.6)	16 (64.0)	23 (88.5)
	Lobular: n (%)	15 (14.4)	5 (19.2)	7 (26.9)	3 (12.0)	0 (0)
	Other: n (%)	14 (13.5)	2 (7.7)	3 (11.5)	5 (20.0)	3 (11.5)
	Not done: n (%)	2 (1.9)	1 (3.8)	0 (0)	1 (4.0)	0 (0)
Tumour subtype	Basal: n (%)	20 (19.2)	3 (11.5)	4 (15.4)	3 (12.0)	9 (34.6)
	LumA: n (%)	51 (49)	11 (42.3)	15 (57.7)	12 (48.0)	13 (50.0)
	LumB: n (%)	33 (31.8)	12 (46.2)	7 (26.9)	10 (40.0)	4 (15.4)
ER status	< 10: n (%)	32 (30.8)	7 (26.9)	7 (26.9)	7 (26.9)	11 (42.3)
	> 10: n (%)	72 (69.2)	19 (73.1)	19 (73.1)	19 (73.1)	15 (57.7)
PR status	< 10: n (%)	47 (45.2)	13 (50.0)	12 (46.2)	10 (38.5)	12 (46.2)
	> 10: n (%)	57 (54.8)	13 (50.0)	14 (53.8)	16 (61.5)	14 (53.8)
Proliferation value (Ki67/Mib1%)	n (missing)	95 (9)	24 (2)	24 (2)	25 (1)	22 (4)
	Mean (Std)	35.3 (25.8)	39.9 (25.0)	28.7 (24.4)	36.3 (24.0)	36.3 (30.3)
	Median (min;max)	30 (1;90)	42.5 (5;90)	17.5 (5;90)	30 (1;90)	30 (3;90)
	Q1, Q3 (IQR)*	12, 50 (38)	17.5, 60 (42.5)	10, 40 (30)	15, 50 (35)	10, 60 (50)
Nodal status	n	104	26	26	26	26
	No: n (%)	41 (39.4)	13 (50)	13 (50)	9 (34.6)	6 (23.1)
	Yes: n (%)	63 (60.6)	13 (50)	13 (50)	17 (65.4)	20 (76.9)

*) Q1 denotes 25% percentile, Q3 denotes 75% percentile, IQR denotes interquartile range

Baseline characteristics of 104 women with breast cancer grouped according to quartiles of the serum-TK1 based cell-loss metric (sTK1, ng x ml^−1^/tumor vol., cm3)

For the 57-patient subgroup baseline demographic data are presented in the additional material (Table A1). The subgroup did not deviate in any respect from the main group.

A general observation was that treatment caused an increase in sTK1 while there was a reduction in tumour volume. Consequently, the cell-loss metric showed a marked increase 48 h after the 2nd treatment cycle (group mean 0.107) compared to baseline (group mean 0.007). Table 2 shows the cell-loss metric in the four groups 48 h after the 2nd cycle; baselines have been subtracted. The metric was 100-fold higher in the quartile-4 group (0.357 units) than in the quartile-1 group (0.004 units). Notably, it was 12-fold higher in the quartile-4 group than in the quartile-3 group (0.03 units) although tumour volumes were similar. The metric of group 4 differed significantly from all other groups (*p* < 0.001).

**Table 2:** Cell-loss metric 48 h after the 2nd cycle of therapy

Statistics	Quartile 1	Quartile 2	Quartile 3	Quartile 4
n	26	26	26	26
Mean (Std)*	0.004 (0.002)	0.010 (0.006)	0.029 (0.010)	0.357 (0.469)
Median (min;max)	0.004 (−0.002;0.008)	0.012 (−0.015;0.017)	0.029 (0.010;0.048)	0.203 (0.048;1.881)
Q1, Q3 (IQR)	0.002, 0.005 (0.003)	0.004, 0.013 (0.005)	0.023, 0.038 (0.015)	0.072, 0.432 (0.36)

*Values are units (sTk1, ng x ml^−1^ / tumor volume, cm^3^)

Descriptive statistics of the TK1-based cell-loss metric 48 h after the 2nd cycle of chemotherapy among 104 women subdivided into four groups according to quartiles of the TK1 cell-loss metric at baseline

Table 3 shows the cell-loss metric in relation to pathological findings. pCR was found in 24 patients (23.1%); remaining tumours of T1 in 38 (36.5%) and of T2/T3 in 42 (40.4%) (for details, see additional material, Table A2). The difference in the cell-loss metric between patients who reached pCR (0.223 units) and those with remaining tumour (0.063 units) was significant (*p* = 0.01).

**Table 3:** Pathologic outcome and cell-loss metric 48 h after the 2nd cycle of therapy

Statistics	pCR*	pT1	pT2 + pT3
n (%)	24 (23.1)	38 (36.5)	42 (40.4)
Mean (Std)**	0.22 (0.47)	0.08 (0.22)	0.05 (0.11)
Median (min;max)	0.06 (0.0;1.87)	0.02 (0;1.25)	0.01 (0.0;0.46)
Q1, Q3 (IQR)	0.02, 0.22 (0.21)	0.01, 0.04 (0.03)	0.004, 0.03 (0.03)

*) pCR denotes pathological complete response in the breast

**) Values are units (sTk1, ng x ml^−1^ / tumor volume, cm^3^)

Descriptive statistics of the TK1-based cell-loss metric 48 h after the 2nd cycle of chemotherapy among 104 women grouped according to pathological status at surgery. The surgery was performed after six cycles of chemotherapy

**Fig. 1:**
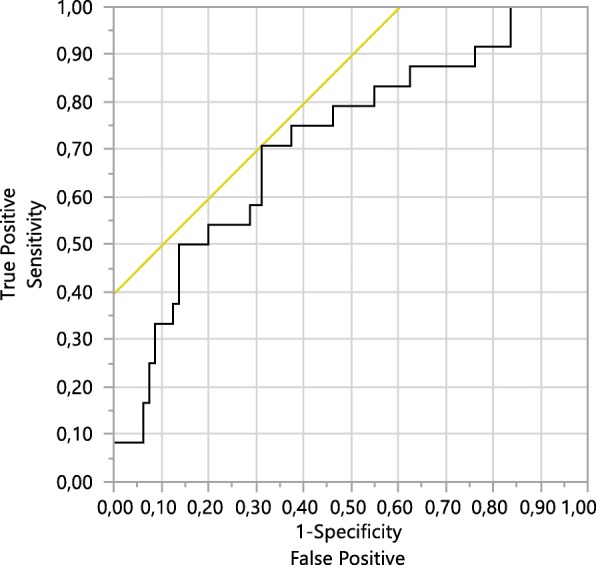
Receiver operating characteristic for distinguishing pCR from remaining tumour in 104 women, based on the cell-loss metric 48 h after the 2nd treatment cycle. At a cut-off value of 0.026 for the cell-loss metric, 1-specificity and sensitivity were 0.31 and 0.71, respectively. ROC Area = 0.714, *p* = 0.02

In a receiver operating analysis for distinguishing pCR from remaining tumour, 1-specifity and sensitivity were 0.31 and 0.71, respectively, at a cut-off value for the cell-loss metric of 0.026(Fig.1).

**Fig. 2:**
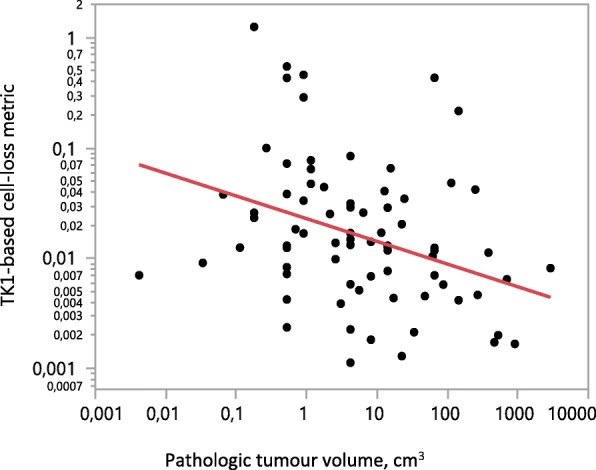
Cell-loss metric 48 h after the 2nd treatment cycle in relation to pathologic tumour volume at surgery after six treatment cycles (*p* = 0.002)

In patients with remaining tumours, tumour volume was inversely related to the cell-loss metric (*p* = 0.002)(Fig.2).

**Fig. 3:**
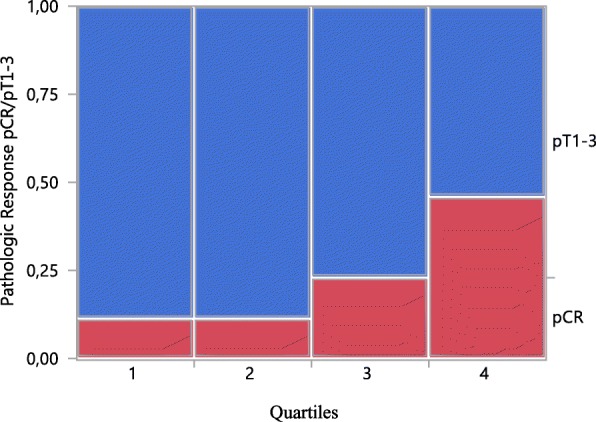
Percentage of pathological complete response in the breast after six cycles of chemotherapy among 104 women, grouped into quartiles according to the cell-loss metric obtained 48 h after the 2nd treatment cycle

The treatment aim to achieve a tumour free breast was reached in 24/104 (23.1%) of the patients. 3/24 cases of pCR were found in each of quartiles 1 and 2, 6/24 in quartile 3, and 12/24 in quartile 4 (Table 4 and Fig.3).

**Table 4:** Baseline cell-loss metric and pathologic outcome

Pathologic status	Quartile 1	Quartile 2	Quartile 3	Quartile 4
	n (%)	n (%)	n (%)	n (%)
pCR*	3 (11.5)	3 (11.5)	6 (23.1)	12 (46.2)
pT1	7 (26.9)	11 (42.3)	13 (50.0)	7 (26.9)
pT2 + pT3	16 (61.5)	12 (46.2)	7 (26.9)	7 (26.9)

*) pCR denotes pathological complete response in the breast

Pathological status among 104 women with breast cancer grouped into four quartiles according to the TK1-based cell-loss metric at baseline

pCR of quartile 1 and 2 differed from those of quartile 4 (*p* = 0.006 and *p* = 0.005, respectively), and the pathological findings of quartile 2 from those of quartile 3 (*p* = 0.029). There was a borderline difference between quartile 3 and 4 (*p* = 0.08).

In the 104 women none of the baseline values was significantly associated with pCR (Table 5).

**Table 5:** Pathologic complete response in relation to baseline variables

Variable	P-value
Stage	0.7427
Pre/Post-menopausal	0.4843
ER < 10>	0.9194
PR < 10>	0.0800
Histological type	0.0989
Lymph nodes	0.2585
Tumour subtype	0.0579
Proliferation value	0.2476

Analysis of variance with pathological complete response in the breast according to baseline variables. Anova with *p*-values for covariates

In order to evaluate the significance of the baseline cell-loss metric for the cell-loss metric established 48 h after cycle 2, all data shown in Tables 2-5 were recalculated but without subtraction of the baselines cell-loss metric (additional material, Tables A3, A4 and A5). The results were very similar, i.e. the proportion of pCR in quartiles 1–4 was 11.5, 11.1, 23 and 48%, respectively. In the analysis of covariates none of all the baseline variables, including the baseline cell-loss metric (*p* = 0.2208), had any significance for the cell-loss metric 48 h after the second cycle of therapy.

Finally, patients were subdivided according to pathologic outcome into pCR and non-pCR. For these two subgroups, tumour volume, and sTK1 per se, and the cell-loss metric were compared at three points in time, namely baseline (*n* = 104), before cycle 2 (*n* = 57), and 48 h after the 2nd cycle (n = 104). The results are shown in Table 6.

**Table 6:** Pathologic response and cell-loss metric at baseline, before and 48 h after the 2nd cycle of therapy

	pCR		non-pCR		
	n	Median (IQR)	n	Median (IQR)	*p*-value
**Baseline**					
Tumour volume	24	80 (137)	80	113 (169)	0.259
sTK1	“	0.35 (0.25)	“	0.30 (0.19)	0.189
sTK1-metric	“	0.0045 (0.0086)	“	0.0027 (0.0043)	**0.030**
**Before cycle 2**					
Tumour volume	15	8 (61)	42	33 (65)	0.944
sTK1	“	0.83 (0.53)	“	0.54 (0.56)	**0.014**
sTK1-metric	“	0.0358 (0.3024)	“	0.0153 (0.0211)	**0.002**
sTK1-metric with baseline subtraction	“	0.0317 (0.2963)	“	0.0117 (0.3706)	**0.003**
**Cycle 2 + 48 h**					
Tumour volume	24	33 (56)	80	48 (65)	0.290
sTK1	“	1.02 (0.64)	“	0.75 (0.65)	0.176
sTK1-metric	“	0.0585 (0.2095)	“	0.0166 (0.0353)	**0.010**
sTK1 metric with baseline subtraction	“	0.0551 (0.2100)	“	0.0128 (0.0318)	**0.003**

Univariate association between pathologic response (pCR, non-pCR) and tumour volume (cm^3^), sTK1 concentration (ng/ml) and sTK1-based cell-loss metric (ng x ml^−1^/cm^3^) at baseline, before cycle 2 and 48 h after cycle 2. *n* = number of patients. Values in bold indicate significance

Notably, in the two groups tumour volume showed a similar (58%) decrease between baseline and as obtained between the 2nd and 3rd cycle, but there was no association between these early measures of tumour volume and pathologic response. However, the cell-loss metric differed significantly between responders and non-responders already at baseline as well as prior to and 48 h after cycle 2. A further observation was the relatively high discriminating power of the cell-loss metric obtained before cycle 2, with positive and negative predictive values of 77.8 and 83.3%, respectively (n = 57). For the metric obtained 48 h after cycle 2, the positive and negative predictive values were 40.5 and 88.7% (*n* = 104).

## Discussion

Like cell proliferation, cell loss plays a significant role in the growth rate of tumours [21]. Both factors contribute to a considerable inter-patient variation in the growth rate of morphologically similar tumours in the same site of the body. In the evaluation of response to therapy, monitoring tumour size via anatomical imaging [11] and molecular imaging, combining tumour size with its metabolism [22], are two frequently used methods.

Here, we evaluated the usefulness of a metric of cell loss, defined as the ratio between the concentration of TK1 in serum and tumor volume, for early prediction of the outcome of chemotherapy in patients with BC. An important finding was that this cell-loss metric, obtained prior to and 48 h after the 2nd cycle of NACT, varied greatly between patients and, in addition, was significantly related to the pathological response established at surgery after 6 cycles of therapy. Thus, for a patient displaying a high cell-loss metric the pathologic response was more favorable. Further, in patients with remaining tumours, tumour size was inversely related to the early cell-loss metric.

These associations between cell-loss and pathologic response are notable not only in the clinical perspective but also because of their biological implications. Firstly, there were substantial inter-patient differences in tumour size prior to treatment, reflecting various stages of development. Also, the change in tumour volume after 6 cycles of therapy differed considerably between patients. In spite of the wide range of tumour size to which sTK1 was related, significant associations were found between the cell-loss metric and the presence or absence of tumour. Secondly, there was a time period of at least 4 months between establishment of the cell-loss metric and surgery. During this interval the patients were subjected to four further treatment cycles, with the addition of bevacizumab. The pathological response is the result of tumour cell loss, which is dependent on the fraction of proliferating cells exposed to varying concentrations of drugs. Tumours may also differ with respect to intrinsic resistance to chemotherapy or in the repopulation capacity of clonogenic cells between the treatment cycles [23]. A poor pathologic response could be due to drug resistance as well as to efficient repopulation between treatments.

Thus, there are several factors that would have the potential of diffusing the association between an early cell-loss metric and the pathologic response. That the early cell-loss metric nevertheless showed a significant relationship with the pathologic response suggests that it represents an inherent tumour property - sensitivity to the cytotoxic substances - that can differ greatly between patients but is comparatively stable within patients, persisting through several cycles of chemotherapy. In fact, also the values of the cell-loss metric established before treatment showed a significant association with the pathologic outcome.

The present findings are also of relevance as regards the mechanisms for release of macromolecules into blood and suggest qualitative differences in cell death between tumours and normal tissues. Normal tissues with high cell turnover are tangibly affected by cytotoxic treatment. In any of the present patients the quantity of normal tissues with high fraction of proliferating cells is likely to have been many times greater than that of the tumour. For instance, the red bone marrow in a woman amounts to approximately 1200 g, containing about 7.5 × 10^11^ nucleated cells [24], 14% being in S-phase [25]. Therefore, if the pathway for removal of damaged cells had been the same in normal tissues and tumour, then the serum level of TK1 would not have been capable of reflecting a property of the tumour. In other words, whereas cell death in tumours is associated with a significant release of TK1, normal tissues must have functions preventing this release. It is generally assumed that the elimination of damaged normal cells follows the apoptotic pathway [26]. Therefore, it seems likely to be a different pathway for tumour cell elimination, namely the necrotic pathway, and this would be responsible for the release of TK1 into blood. Leakage of macromolecules via the necrotic pathway is believed to be related to active phagocytosis [27]. This makes it tempting to reflect upon certain new concepts of regulated immunity in oncology as well as the results of immunotherapy by blockade of the CTLA-4 protein [28] or PD-1 protein [29] on the surface of T-cells. Possibly, the success of such enhanced phagocytosis could be monitored via measurements of the concentration of TK1 in serum.

In 57 of the patients, the cell-loss metric could be established also prior to the 2nd treatment. Although the values 48 h after treatment were approximately 50% greater, it appears that the relationship with pathologic response was higher for the pre-treatment values. An explanation for this could be that during treatment cell loss in normal tissues temporarily exceeds the capacity of the apoptotic pathway, resulting in a non-tumour specific release of TK1 into blood. Such a confounding factor would be less pronounced 2–3 weeks after treatment. As regards other tumour- or patient-related data, we did not find any factors which correlate with, or explain, the cell-loss metric. The values 48 h after the 2nd treatment were independent of the baseline. In addition, the prediction of pathologic response could not be improved by combining the cell-loss metric with the histologic proliferation marker Ki67/Mib1.

It might appear remarkable that such a basic and well-established tumour property as the fraction of proliferating cells did not contribute to the predictive power of the cell-loss metric. Nevertheless, there is a reasonable explanation for this finding. Proliferation and cell loss are both complex phenomena. Proliferation may constitute a primary component in a network of processes whereby cytotoxic therapy results in cell loss. In other words, cell loss would be determined not only by the fraction of proliferating cells (as expressed by Ki67/Mib1) but also by a multitude of less well-known factors. If the cell-loss metric thus reflects a sum effect of several mechanisms, including the rate of proliferation, then, adding Ki67/Mib1 would not contribute to the predictive value of the metric. In the practical perspective, the cell-loss metric might be considered causally closer to the outcome of treatment.

The finding that a number of tumour properties did not differ between the quartile groups does not imply that they are clinically insignificant but that they are independent of the cell-loss metric. Therefore, it is logically possible that some of them would improve the prediction of pathologic response. This is the main theme of a following study (to be published), where it was found that combining the cell-loss metric with histopathologic markers, such as receptors for oestrogen and progesterone, improves the predictive power in terms of both sensitivity and specificity.

The clinical value of tumour biomarkers is to guide therapy. A distinction is made between prognostic markers, supposed to provide information about long-term outcome, and predictive markers, which reveal a tumour’s response to treatment. Ideally, the adequate choice of therapy would be based on tumour or patient characteristics established before treatment. For a defined type of tumour there is, nevertheless, always an inter-patient variability in the response to treatment. Therefore, predictive markers for early detection of the effects of treatment would be a valuable complement to tumour characteristics established at diagnosis. Among the most well-established tissue markers in oncology are the receptors for oestrogen, progesterone and growth-factor 2 [30]. These are all used in the primary characterization of BC and constitute the targets in hormone therapy as well as in treatment with monoclonal antibodies. Molecular characterization of tumours has generated an increasing number of putative predictive biomarkers [9, 10]. The manifold of such markers is in line with the demands of a more individualized treatment. In addition, the increasing sub-classification of tumours requires principles for exploring the usefulness of new biomarkers.

Nevertheless, there is a paucity of methods for the early evaluation of tumour response during treatment. Such methods would give a valuable contribution particularly in the management of patients for whom the statistically calculated benefit of a standard treatment is low and has to be balanced against unnecessary side effects. For instance, in low-grade, low-stage ER+/HER-2neu luminal-A tumours, pCR after cytotoxic treatment was achieved in less than 10% of patients and, in addition, pCR was not prognostic for long-term survival [1, 2]. Early identification of individual patients with poor response would permit a switch to hormone therapy or motivate immediate surgery - and suffering due to unnecessary side effects could be avoided. In BC, clinical monitoring of tumour volume early during treatment have motivated shifts from anthracycline-based therapy to docetaxel [31] and from docetaxel-doxorubicin-cyclophosphamide to vinorelbine-capecitabine [32] in non-responding patients; and these shifts in treatment were associated with enhanced clinical and pathological remissions.

A few studies deal with the release of macromolecules early during chemotherapy and how such early response markers are associated with pathologic outcome or long-term survival. In patients with lung cancer a high activity of TK1 in serum after the first and second cycles of cytotoxic treatment was associated with a significantly longer survival [33]. Analogously, in colon cancer a lack of increased TK1 activity during chemotherapy was related to a poor prognosis [34]. Further, during chemotherapy for colon cancer, patients in whom the concentrations of cell-free mutated DNA had declined dramatically prior to the second treatment also displayed a substantial reduction in radiologic measures of tumour volume [35]. In lung cancer, a rapid decrease in the serum concentration of mutated *EGFR*-DNA 14 days after initiating treatment with erlotinib (a tyrosine kinase inhibitor) was associated with tumour shrinkage 2 months later [36]. Likewise, during the first week of chemotherapy for lung cancer, the levels of nucleosomes were substantially lower in patients who responded to treatment than in non-responders [37].

In BC, no significant changes in nucleosome levels have been found during the first two treatment cycles of NACT [38]. However, an increased concentration of uncleaved cytokeratin-18, which is an indicator of necrotic cell death, early during the first cycle was associated with a favorable clinical response and improved survival [39]. In triple-negative non-metastatic BC, the persistence of TP53 mutated DNA in serum before the 2nd cycle of anthracycline/taxane-based chemotherapy has been related to a shorter disease-free and overall survival. However, no association was found between ctDNA levels and pCR [40]. In a pioneering study, patients with metastatic BC who displayed persistent high levels of circulating tumour cells after 3 weeks of cytotoxic therapy were subjected to a shift to another drug; there was, however, no improvement in survival [41].

To our knowledge there are no studies which address the clinical value of a measure that relates the levels of a macromolecule, released from disrupting tumour cells, to the volume of the tumour. The usefulness and predictive power of the TK1-based cell-loss metric have the potential of being improved in several ways. A limitation of the present study was that the patients were examined and treated in five different clinics. Methods for estimating tumour size included caliper measurement, mammography and ultrasonography, the accuracy of which ranges between 57 and 79% [42]. Methods may differ not only in accuracy but also with respect to the smallest tumour that can be detected. Thus, it might be considered whether in cases with small tumours a less sophisticated method would tend to yield values close to zero and, hence, a converse bias in the cell-loss metric. In the present study, the distribution of data does not suggest any bias of this kind. Nevertheless, although routine clinical management permits a variety of techniques for measuring tumour volume, new prognostic tools may motivate more standardized and accurate methods. Magnetic resonance imaging would have provided a higher accuracy and consistency in data, particularly in cases where tumours were small already prior to treatment. Another strategy for improving sensitivity and accuracy is to combine two different methods. At the Karolinska University Hospital, were the majority of the present material was handled, each patient was routinely examined with both mammography and ultrasonography.

Reactions of lymph nodes on therapy could not be assessed, but release of TK1 from metastatic lymph nodes cannot be excluded. Another issue is the time point for establishing the cell-loss metric. The precise time course for treatment-induced changes in sTK1 remains to be clarified, and it may, in addition, be dependent on the type of treatment. As already noted, the predictive value of the cell-loss metric appears to be higher prior to the 2nd treatment than 48 h after treatment. Advantages of the present study were the prospective layout of the original clinical trial and the absence of patients with distant metastases, which would have constituted sources of TK1 with unknown volumes. Prospective studies should be performed to confirm the present findings, to establish the optimal time points for the cell-loss metric during different treatments, and to define cut-off values for discriminating between responders and non-responders.

## Conclusions

The present study introduces a measure of cell loss, obtained by combining the serum level of TK1, released from disrupted tumour cells, with tumour volume. Established early during chemotherapy, this metric showed a considerable inter-patient variability and a significant association with later pathologic response. Thus, it appears to reflect an inherent property of the tumour, of importance for tumour growth and response to treatment. In the practical perspective, monitoring treatment response by means of the cell-loss metric could be valuable in individualized therapy as well as in the development of new cytotoxic drugs or targeted therapies.

## Supplementary information

**Additional file 1.** Flow chart

**Additional file 2: Table A1.** Baseline characteristics for the subgroup of 57 women.

**Additional file 3: Table A2.** Pathologic findings in the breast and axillary lymph nodes

**Additional file 4: Table A3.** Cell-loss metric 48 h after the 2nd cycle of therapy without baseline subtraction

**Additional file 5: Table A4.** Pathologic outcome and cell-loss metric 48 h after the 2nd cycle of therapy without baseline subtraction

**Additional file 6: Table A5.** Baseline cell-loss metric and pathologic outcome

**Additional file 7: Table A6.** Pathologic complete response in relation to baseline variables

## Abbreviations

BC: Breast cancer
NACT: Neoadjuvant chemotherapy
pCR: pathologic complete response
sTK1: serum thymidine kinase1 concentration
TK1: Thymidine kinase 1
