# Supplementary material for: Early prediction of pathologic response to neoadjuvant treatment of breast cancer: use of a cell-loss metric based on serum thymidine kinase 1 and tumour volume
Source: BMC Cancer. 2020 May 18;20:440. doi: 10.1186/s12885-020-06925-y (PMC7236455; doi:10.1186/s12885-020-06925-y)
Supplement: Supplementary file 1 — Additional file 1. Flow chart [file 12885_2020_6925_MOESM1_ESM.docx]

**Flow chart**

Study start

n=150

n=148

n=2

Death from toxicity refused participation

Missing sTK 1 value at baseline (but not volume)

n=139

n=9

n=132

n=7

Missing volume at baseline (but not sTK1)

Missing both sTK 1 and volume at cycle 2

n=127

n=5

Missing sTK 1 value at cycle 2 (but not volume)

n=116

n=11

Missing volume at cycle 2 (but not sTK1)

n=12

n=104

Final data

n=104

Missing data of initially 150 patients with breast cancer in the analysis of the sTK1 cell-loss metric 48h after the 2^nd^ cycle of neoadjuvant chemotherapy
