# Supplementary material for: Early prediction of pathologic response to neoadjuvant treatment of breast cancer: use of a cell-loss metric based on serum thymidine kinase 1 and tumour volume
Source: BMC Cancer. 2020 May 18;20:440. doi: 10.1186/s12885-020-06925-y (PMC7236455; doi:10.1186/s12885-020-06925-y)
Supplement: Supplementary file 2 — Additional file 2: Table A1. Baseline characteristics for the subgroup of 57 women. [file 12885_2020_6925_MOESM2_ESM.docx]

**Table A1 Baseline characteristics for the subgroup of 57 women.**

| Variable | Statistics | Total |
| --- | --- | --- |
| Age at registration | n | 57 |
|  | Mean (Std) | 49.0 (9.4) |
|  | Median (min;max) | 48.8 (33.1;66.0) |
| Menopause | Post: n (%) | 22 (38.6) |
|  | Pre: n (%) | 35 (61.4) |
| Stage | 1: n (%) | 2 (3.5) |
|  | 2: n (%) | 21 (36.8) |
|  | 3: n (%) | 34 (59.7) |
| Tumour volume, cm3 | n | 57 |
|  | Mean (Std) | 187 (415) |
|  | Median (min;max) | 97 (4;3052) |
| sTK1 ng/ml | n | 57 |
|  | Mean (Std) | 0.329 (0.140) |
|  | Median (min;max) | 0.295 (0.1;0.72) |
| Cell-loss metric, units | n | 57 |
|  | Mean (Std) | 0.009 (0.015) |
|  | Median (min;max) | 0.003 (0.001;0.069) |
| Histological type | Ductal: n (%) | 38 (66.7) |
|  | Lobular: n (%) | 7 (12.3) |
|  | Other: n (%) | 10 (17.5) |
|  | Not done: n (%) | 2 (3.5) |
| Tumour subtype | Basal: n (%) | 12 (21.0) |
|  | LumA: n (%) | 27 (47.4) |
|  | LumB: n (%) | 18 (31.6) |
| ER status | < 10: n (%) | 19 (33.3) |
|  | >10: n (%) | 38 (66.7) |
| PR status | < 10: n (%) | 22 (38.6) |
|  | >10: n (%) | 35 (61.4) |
| Proliferation value (Ki67/Mib1%) | n (missing) | 54 (3) |
|  | Mean (Std) | 36 (26.9) |
|  | Median (min;max) | 30 (1;90) |
| Nodal status | n | 57 |
|  | No: n (%) | 21 (36.8) |
|  | Yes: n (%) | 36 (63.2) |
