# Supplementary material for: Early prediction of pathologic response to neoadjuvant treatment of breast cancer: use of a cell-loss metric based on serum thymidine kinase 1 and tumour volume
Source: BMC Cancer. 2020 May 18;20:440. doi: 10.1186/s12885-020-06925-y (PMC7236455; doi:10.1186/s12885-020-06925-y)
Supplement: Supplementary file 3 — Additional file 3: Table A2. Pathologic findings in the breast and axillary lymph nodes [file 12885_2020_6925_MOESM3_ESM.docx]

**Table A2. Pathologic findings in the breast and axillary lymph nodes**

|  | ypT0 | ypT1 | ypT2 | ypT3 |
| --- | --- | --- | --- | --- |
| ypN0 (%) | 12 (50) | 16 (42) | 5 (19) | 1 (7) |
| ypN1 (%) | 7 (29) | 13 (34) | 13 (48) | 5 (33) |
| yp >N1 (%) | 5 (21) | 9 (24) | 9 (33) | 9 (60) |
| Total | 24 | 38 | 27 | 15 |

Pathological findings in the breast and numbers of axillary lymph nodes in 104 patients after 6 treatment cycles.
