# Supplementary material for: Early prediction of pathologic response to neoadjuvant treatment of breast cancer: use of a cell-loss metric based on serum thymidine kinase 1 and tumour volume
Source: BMC Cancer. 2020 May 18;20:440. doi: 10.1186/s12885-020-06925-y (PMC7236455; doi:10.1186/s12885-020-06925-y)
Supplement: Supplementary file 4 — Additional file 4: Table A3. Cell-loss metric 48 h after the 2nd cycle of therapy without baseline subtraction [file 12885_2020_6925_MOESM4_ESM.docx]

**Table A3. Cell-loss metric 48h after the 2^nd^ cycle of therapy without baseline subtraction**

| Statistics | Quartile 1 | Quartile 2 | Quartile 3 | Quartile 4 |
| --- | --- | --- | --- | --- |
| n | 26 | 26 | 26 | 26 |
| Mean (Std) | 0.01 (0.00) | 0.01 (0.00) | 0.04 (0.01) | 0.38 (0.47) |
| Median (min;max) | 0.01 (0.00;0.01) | 0.01 (0.01;0.02) | 0.03 (0.02;0.05) | 0.22 (0.06;1.89) |
| Q1, Q3 (IQR) | 0.003, 0.01 (0.00) | 0.01, 0.02 (0.01) | 0.03, 0.04 (0.02) | 0.09, 0.45 (0.37) |

Descriptive statistics for the cell-loss metric 48h after the 2^nd^ treatment cycle; baseline not subtracted. The 104 women have been subdivided into quartiles according to the baseline cell-loss metric.
