# Supplementary material for: Early prediction of pathologic response to neoadjuvant treatment of breast cancer: use of a cell-loss metric based on serum thymidine kinase 1 and tumour volume
Source: BMC Cancer. 2020 May 18;20:440. doi: 10.1186/s12885-020-06925-y (PMC7236455; doi:10.1186/s12885-020-06925-y)
Supplement: Supplementary file 5 — Additional file 5: Table A4. Pathologic outcome and cell-loss metric 48 h after the 2nd cycle of therapy without baseline subtraction [file 12885_2020_6925_MOESM5_ESM.docx]

**Table A4**. **Pathologic outcome and cell-loss metric 48h after**

**the 2^nd^ cycle of therapy without baseline subtraction**

| Statistics | pCR | pT1-pT3 |
| --- | --- | --- |
| n | 24 | 80 |
| Mean (Std) | 0.24 (0.48) | 0.07 (0.17) |
| Median (min;max) | 0.06 (0;1.89) | 0.02 (0;1.26) |
| Q1, Q3 (IQR) | 0.02, 0.22 (0.2) | 0.01, 0.04 (0.03) |

Descriptive statistics for the cell-loss metric 48h after the 2^nd^ treatment cycle; baseline not subtracted. The 104 women have been subdivided according to pathologic outcome after 6 treatment cycles.
