# Supplementary material for: Early prediction of pathologic response to neoadjuvant treatment of breast cancer: use of a cell-loss metric based on serum thymidine kinase 1 and tumour volume
Source: BMC Cancer. 2020 May 18;20:440. doi: 10.1186/s12885-020-06925-y (PMC7236455; doi:10.1186/s12885-020-06925-y)
Supplement: Supplementary file 6 — Additional file 6: Table A5. Baseline cell-loss metric and pathologic outcome [file 12885_2020_6925_MOESM6_ESM.docx]

**Table A5 Baseline cell-loss metric and pathologic outcome**

| Pathologic status | Quartile 1  n (%) | Quartile 2  n (%) | Quartile 3  n (%) | Quartile 4  n (%) |
| --- | --- | --- | --- | --- |
| pCR | 3 (11.5) | 3 (11.5) | 6 (23) | 12 (46) |
| pT1 - pT3 | 23 (88.5) | 23 (88.5) | 20 (77) | 14 (54) |

Pathologic outcome among 104 women, subdivided into quartiles according to baselines for the cell-loss metric.
