# Supplementary material for: Early prediction of pathologic response to neoadjuvant treatment of breast cancer: use of a cell-loss metric based on serum thymidine kinase 1 and tumour volume
Source: BMC Cancer. 2020 May 18;20:440. doi: 10.1186/s12885-020-06925-y (PMC7236455; doi:10.1186/s12885-020-06925-y)
Supplement: Supplementary file 7 — Additional file 7: Table A6. Pathologic complete response in relation to baseline variables [file 12885_2020_6925_MOESM7_ESM.docx]

**Table A6. Pathologic complete response in relation to**

**baseline variables**

| Variable | P-value |
| --- | --- |
| Stage | 0.6489 |
| Pre/Post- menopausal | 0.5230 |
| ER BL <10> | 0.8227 |
| PR BL <10> | 0.0856 |
| Histological type | 0.1269 |
| Lymph nodes | 0.2341 |
| Tumour subtype | 0.1012 |
| Proliferation value | 0.3535 |
| Cell-loss metric | 0.2208 |

Association between pathologic complete

response and baseline variables in 104 patients.
